# VANGL2 regulates luminal epithelial organization and cell turnover in the mammary gland

**DOI:** 10.1038/s41598-019-43444-8

**Published:** 2019-05-08

**Authors:** Prestina Smith, Nathan Godde, Stefany Rubio, Melawit Tekeste, Eszter K. Vladar, Jeffrey D. Axelrod, Deborah J. Henderson, Michal Milgrom-Hoffman, Patrick O. Humbert, Lindsay Hinck

**Affiliations:** 1 Department of Molecular, Cell and Developmental Biology, University of California, Santa Cruz, CA 95064 USA; 2 Cell Cycle and Cancer Genetics Laboratory, Peter MacCallum Cancer Centre, St Andrews Place, Melbourne, VIC 3002 Australia; 3 Sir Peter MacCallum Department of Oncology, University of Melbourne, Parkville, VIC 3010 Australia; 4 Australian Animal Health Laboratory, Commonwealth Scientific and Industrial Research Organisation (CSIRO), Victoria, 3220 Australia; 5 Department of Pathology, Stanford University School of Medicine, Stanford, CA 94305 USA; 6 Division of Pulmonary Sciences and Critical Care Medicine, Department of Medicine and Department of Cell and Developmental Biology, University of Colorado Denver School of Medicine, Aurora, CO 80045 USA; 7 Cardiovascular Research Centre, Institute of Genetic Medicine, Newcastle University, Newcastle upon Tyne, UK; 8 Department of Biochemistry & Genetics, La Trobe Institute for Molecular Science, La Trobe University, Melbourne, Victoria 3086 Australia; 9 Department of Biochemistry & Genetics, University of Melbourne, Parkville, VIC 3010 Australia; 10 Department of Clinical Pathology, University of Melbourne, Parkville, VIC 3010 Australia

**Keywords:** Body patterning, Mammary stem cells

## Abstract

The VANGL family of planar cell polarity proteins is implicated in breast cancer however its function in mammary gland biology is unknown. Here, we utilized a panel of *Vang1* and *Vangl2* mouse alleles to examine the requirement of VANGL family members in the murine mammary gland. We show that *Vang1CKO*^*Δ/Δ*^ glands display normal branching while *Vangl2*^*flox/flox*^ and *Vangl2*^*Lp/Lp*^ tissue exhibit several phenotypes. In *MMTV*-*Cre;Vangl2*^*flox/flox*^ glands, cell turnover is reduced and lumens are narrowed. A *Vangl2* missense mutation in the *Vangl2*^*Lp/Lp*^ tissue leads to mammary anlage sprouting defects and deficient outgrowth with transplantation of anlage or secondary tissue fragments. In successful *Vangl2*^*Lp/Lp*^ outgrowths, three morphological phenotypes are observed: distended ducts, supernumerary end buds, and ectopic acini. Layer specific defects are observed with loss of *Vangl2* selectively in either basal or luminal layers of mammary cysts. Loss in the basal compartment inhibits cyst formation, but has the opposite effect in the luminal compartment. Candidate gene analysis on *MMTV*-*Cre;Vangl2*^*flox/flox*^ and *Vangl2*^*Lp/Lp*^ tissue reveals a significant reduction in *Bmi1* expression, with overexpression of *Bmi1* rescuing defects in *Vangl2* knockdown cysts. Our results demonstrate that VANGL2 is necessary for normal mammary gland development and indicate differential functional requirements in basal versus luminal mammary compartments.

## Introduction

Recent studies have implicated the planar cell polarity (PCP) genes *VANGL1* and *VANGL2* in breast cancer^1^. A high level of VANGL1 expression is associated with poor prognosis and relapse in breast cancer patients^2^. Similarly, upregulation of VANGL2 was identified in the more aggressive basal type tumors and is also associated with poor prognosis^3^. While alterations of VANGL1 and VANGL2 in breast cancer have been investigated, their function in normal breast development is still unknown. Here we provide the first analysis of VANGL function in mammary gland development *in vivo*.

The mammary gland is a branched organ with epithelial ducts that grow outward from the nipple into a surrounding fat pad. These ducts are bi-layered structures, composed of an outer basal layer of contractile myoepithelial cells and an inner luminal layer of secretory cells. Mammary identity is established early in the developing embryo. By E11.5, FGF and WNT signals drive cell fate specification in order to produce the first mammary epithelium^4^. This group of cells further expands around E15.5 to form a mammary bud. At birth, the mammary gland exists as a rudimentary tree with few branches and a hollow lumen. The mammary gland then remains quiescent until puberty when it undergoes massive growth by branching. During branching morphogenesis, end buds (EBs) establish the mammary tree through two types of branching. Initially, a small number of terminal EBs (TEBs) sprout into the fat pad and, through coordinated migration and bifurcation events, produce primary branches that extend from the nipple to the outer edges of the fat pad. Next, lateral end buds form on the sides of primary branches and sprout secondary and tertiary branches. The formation and expansion of EBs in the mammary gland relies on the precise deployment of extracellular signals. One of the major drivers of mammary gland morphogenesis is WNT signaling^5^.

WNT signaling can be divided into canonical and non-canonical pathways. Canonical WNT signaling has been implicated in multiple aspects of mammary gland development^5^, however, details regarding non-canonical WNT signaling in the mammary gland remain elusive. The non-canonical WNT/Planar cell polarity (PCP) signaling axis propagates directional information across sheets of epithelium. At the core of PCP signaling is the Van Gogh Like (VANGL) family of transmembrane proteins: VANGL1 and VANGL2^6^. One of the VANGL family proteins, VANGL2, is required for proper branching and tubulogenesis of the kidney and lung, but the exact mechanism of this action is unknown^7–11^.

To examine the requirement of VANGL family members in the murine mammary gland, we utilized a panel of *Vang1 and Vangl2* mouse alleles. Here, we report that *Vangl2* missense and loss-of-function mutations stunt mammary gland development whereas a *Vangl1* hypomorphic mutation does not affect mammary outgrowth or branching morphogenesis. In addition, using different *Vangl2* alleles, we demonstrate that loss of cell surface VANGL2 results in different phenotypes compared to *Vangl2* deletion. Using *in vitro* primary cultures, we show that VANGL2 has distinct functions in the basal and luminal cell compartments. Finally, we show that loss of *Vangl2* lowers expression of the polycomb group repressor *Bmi1* and hinders cyst formation, while overexpression of the gene rescues cyst formation *in vitro*. Our studies provide insight into mechanisms that control growth and development in the mammary gland and show a novel mechanism for PCP signaling in influencing transcriptional repressors. Our studies also further broaden the scope of non-canonical WNT signaling in the mammary gland and draw useful comparisons between different *Vangl* loss-of-function models.

## Results

### *Vangl2* is expressed in multiple cell populations in the mammary gland

**Figure 1:**
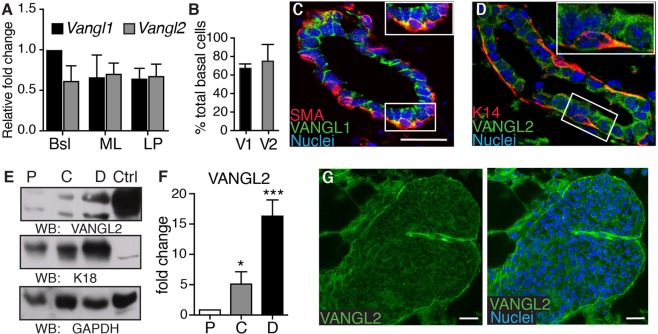
*Vangl1* and *Vangl2* expression in the mammary gland. (**A**) RT-qPCR analysis of *Vangl1* and *Vangl2* mRNA levels in FACS-purified basal (Bsl), mature luminal (ML), and luminal progenitor (LP) cells (n = 3). (**B**) Quantification of basal cells positive for VANGL1 (V1) or VANGL2 (V2) by immunofluorescence in mature virgin glands. Immunostained *WT* 8 weeks old mammary tissue shows levels of VANGL1 (green) with Smooth Muscle Actin (SMA)(red), and (**D**) VANGL2 (green) with Cytokeratin 14 (K14)(red). (**E**,**F**) Representative immunoblots (**E**) and quantification (**F**) of VANGL2, Cytokeratin 18 (K18) and GAPDH (control) in proximal (P), Central (**C**) and Distal (**D**) regions of 8 weeks old mammary gland. HEK293 lysate was used as the control (Ctrl) sample (n = 3). (**G**) Immunostained 5.5 weeks old *WT* gland shows VANGL2 (green) in a bifurcating TEB (nuclei, blue). Data are represented as mean +/− SEM. Scale bars represent 20 μm. Two way ANOVA *p < 0.05 and ***p < 0.001.

To establish the role of PCP genes *Vangl1* and *Vangl2* in the mammary gland, we initially examined their mRNA levels using RT-qPCR. Cells isolated from mammary glands harvested from adult wildtype (*WT*) mice were FACS purified into three subpopulations: basal (Lin-CD24^+^CD29^hi^), mature luminal (Lin-CD24^lo^CD29^+^CD61^−^), and luminal progenitor (Lin-CD24^lo^CD29^+^CD61^+^). RNA isolated from each cell type was subjected to RT-qPCR using primers specific to *Vangl1* and *Vangl2*. Results from these experiments show *Vangl1* and *Vangl2* expressed in all mammary cell populations (Fig. 1A). Re-analysis of a previously published GEO dataset (GSE19446)^12^ that profiled FACS sorted normal mouse mammary cell subpopulations independently supported this observation (Supp. Fig. 1).

Previous studies have linked the function of VANGL1 and VANGL2 to their subcellular localization, and their role in PCP is characterized by their membrane localization at the apical epithelial cell junctions and within recycling endosomes^13^. In order to better understand these proteins in the mammary gland, we investigated the subcellular localization of the VANGL proteins by immunohistochemical analysis of sectioned mammary glands from mature virgin mice stained with antibodies generated against VANGL1, VANGL2, and basal lineage markers K14 and SMA. Consistent with the mRNA expression, VANGL1 and VANGL2 were expressed in all luminal cells and approximately 70% of basal cells (Fig. 1B). Within each cell, VANGL1 and VANGL2 were found both at the membrane, consistent with their role in cell/cell interactions, and in a punctate pattern within the cytoplasm, consistent with their active regulation by endocytosis (Fig. 1C,D)^13^.

Previous studies have shown the importance of graded VANGL2 expression during tissue morphogenesis^14^. To further investigate the role of VANGL2 in the adult mammary gland, we quantified protein levels across the gland. To this end, we harvested mammary glands from *WT* mature virgin mice, cut the tissue into the proximal (near nipple, P), central (C) and distal (TEB-containing, D) regions and used protein isolated from each region for immunoblotting with antibodies against VANGL2, K18 (luminal cells) and GAPDH (loading control). We found VANGL2 present in a gradient from the nipple to the TEBs, with a 5-fold VANGL2 increase in the central and a 15-fold increase in the distal, compared to the proximal, regions (Fig. 1E,F, Supp. Fig. 4). To examine VANGL2 subcellular localization in distal TEBs, where it is more concentrated, we micro-dissected them and immunostained with anti-VANGL2 antibodies. We observed VANGL2 staining in a punctate pattern in the cytoplasm and at the membrane in the unpolarized luminal cells (body cells) that constitute the body of the end bud (Fig. 1G), suggesting a role for VANGL2 in TEBs during ductal morphogenesis. Taken together, these results show both VANGL1 and VANGL2 are expressed in mammary epithelial cells and suggest a role for the proteins in the development of the mammary gland.

### Vangl1 is dispensable for normal mammary morphogenesis

**Figure 2:**
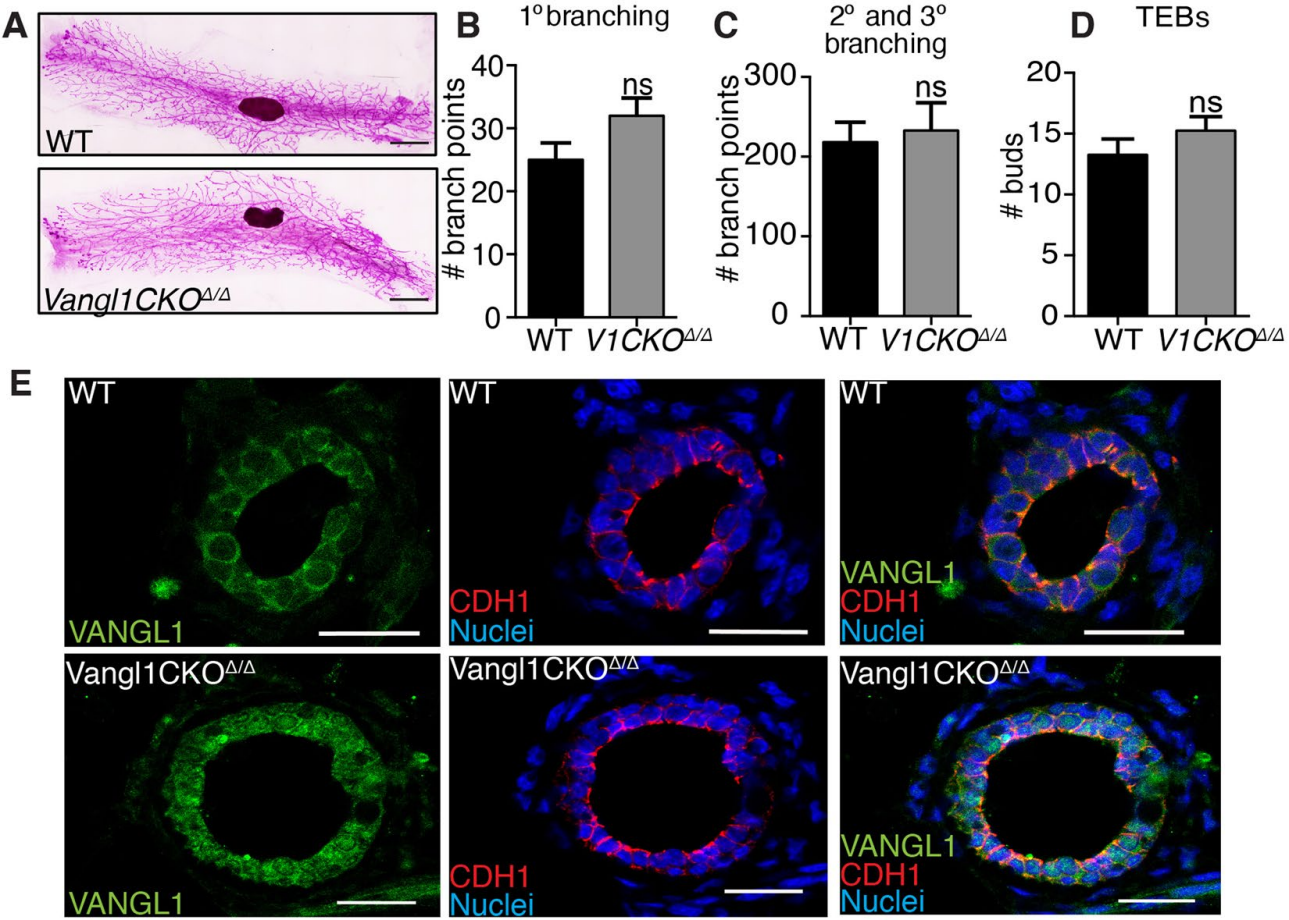
Loss of VANGL1 does not affect mammary growth or branching. (**A**) Representative images of ductal outgrowth in carmine stained *WT* and *Vangl1CKO*^Δ/Δ^ mammary glands at 8 weeks. Quantification of primary (**B**), secondary and tertiary branching (**C**) and TEBs (**D**) (n = 3 mice per genotype). (**E**) Immunostaining tissue from *WT* (top) and *Vangl1CKO*^Δ/Δ^ (bottom) mammary glands from 8 weeks old mice shows localization of VANGL1 (green) on the cell borders (*WT*) or in a punctate pattern in the cytosol (*Vangl1CKO*^Δ/Δ^) of mammary epithelial cells (left), E-cadherin (CDH1) (red) on cell borders of both *WT* and *Vangl1CKO*^Δ/Δ^ tissue (middle), and merged VANGL1 and E-cadherin (right). Data are represented as mean +/− SEM. Scale bars represent 1.5 mm (**A**) and 20 μm (**E**). Student’s t-test ns = not significant.

VANGL1 and VANGL2 are transmembrane proteins that rely on cell surface expression to carry out PCP signaling^15–17^. In order to investigate the role of VANGL1 and VANGL2 in the mammary gland, we examined the loss-of-function phenotypes of these proteins using mice carrying mutations that disrupt trafficking of the proteins to the cell surface. The previously described *Vangl1CKO* mice^15^ were generated by using the HRPT-Cre allele to delete all of the transmembrane domains of *Vangl1* in the germline. Consequently, these homozygous deleted mice (*Vang1CKO*^*Δ/Δ*^) produce a VANGL1 protein that is improperly trafficked to the membrane, disrupting the function of VANGL1 in PCP. To investigate the overall structure of *Vangl1CKO*^*Δ/Δ*^ mammary glands, we carmine stained whole mounted glands from mature virgin mice and observed a fully expanded mammary epithelial tree, characterized by ducts reaching the outer edges of the fat pad in a growth pattern similar to *WT* (Fig. 2A). We performed branching analysis to assay TEB bifurcation (primary branching) and secondary/tertiary branching and discovered no significant difference between *WT* and *Vangl1CKO*^*Δ/Δ*^ tissue (Fig. 2B–D). We further investigated the role of VANGL1 in mammary morphogenesis by examining cell morphology in *Vangl1CKO*^*Δ/Δ*^ glands. To confirm mislocalization of VANGL1, we immunostained with anti-VANGL1 and observed an increase in puncta localized in the cytoplasm of *Vangl1CKO*^*Δ/Δ*^ tissue (Fig. 2E). This observation was consistent with a previously described trafficking defect^15^. Additionally, we immunostained with anti-CDH1 and observed a normal epithelial staining pattern in both *WT* and *Vangl1CKO*^*Δ/Δ*^ tissue, with E-cadherin localized to cell junctions (Fig. 2E). Together, these data suggest that VANGL1 signaling at the plasma membrane is dispensable in mammary development.

### Loss of *Vangl2* impairs cell turnover and generates compacted ducts with narrow lumens

**Figure 3:**
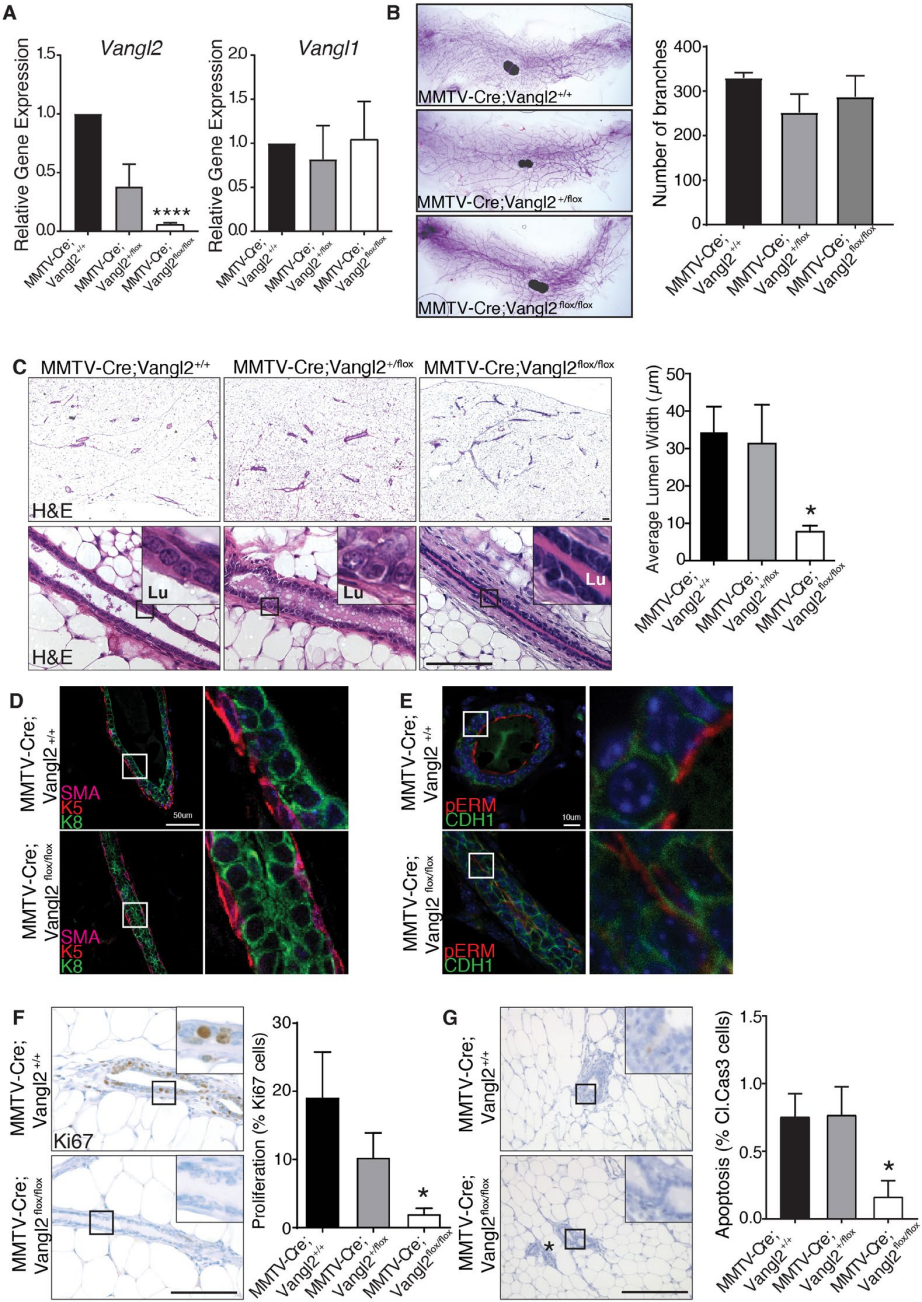
Deletion of Vangl2, but not Vangl1, in the mammary gland results in narrow ducts and low cell turnover. (**A**) RT-qPCR analysis of *Vangl1* and *Vangl2* mRNA levels in the mammary glands of *MMTV*-*Cre*, *MMTV*-*Cre;Vangl2*^*flox/*+^ and *MMTV*-*Cre;Vangl2*^*flox/flox*^ at 10 weeks of age. (**B**) Representative images of 10 week old, carmine stained mammary glands and quantification of total branch number from *MMTV*-*Cre*, *MMTV*-*Cre;Vangl2*^*flox/*+^ and *MMTV*-*Cre;Vangl2*^*flox/flox*^ mice (n = 3 mice per genotype). (**C**) Histological analysis by H&E staining show normal duct formation in 10 week old *MMTV*-*Cre* and *MMTV*-*Cre;Vangl2*^*flox*/+^ glands, whereas ducts from *MMTV*-*Cre;Vangl2*^*flox/flox*^ glands are narrow and have significantly diminished lumens (n = 3 mice/genotype). (**D**) Immunostaining in mammary ducts show normal distribution of luminal (Cytokeratin 8 (K8), green), and basal (K5), red; SMA magenta) cell populations. (**E**) Immunofluorescence of adhesion junctional protein E-Cadherin (CDH1, green) and apical membrane marker pERM (red). (**F**,**G**) Immunostaining in 10 week old *MMTV*-*Cre;Vangl2*^+*/*+^ and *MMTV*-*Cre;Vangl2*^*flox/flox*^ glands and quantitation of (**F**) Ki67 and (**G**) Cleaved Caspase 3 (n = 3–5 mice per genotype). Lu indicates lumen. * indicates non-magnified duct. Data are represented as mean +/− SEM. Scale bar represents 100 μm (**C**,**F**,**G**) 50 μm (**D**) and 10 μm (**E**). Student’s t-test *p < 0.05 and ****p < 0.0001.

To investigate the consequences of *Vangl2* deletion in the mammary gland in the context of normal *Vangl1* expression, we utilized a previously developed floxed allele of *Vangl2* consisting of loxP sites flanking exon 4^18^. Expression of Cre in these mice introduces a premature stop codon and subsequent loss of all four transmembrane domains and the C-terminal PDZ-binding motif. To specifically delete *Vangl2* in the mammary epithelium, we crossed these mice with *MMTV*-*Cre* transgenic mice that delete floxed genes in both luminal and basal mammary epithelial cells^19^. We then confirmed dose dependent loss of *Vangl2*, but not *Vangl1*, in the mammary glands of *MMTV*-*Cre;Vangl2*^*flox/*+^ and *MMTV*-*Cre;Vangl2*^*flox/flox*^ mice by RT-qPCR (Fig. 3A). To assess the developmental impact of VANGL2 loss on branching, we examined whole mount preparations of inguinal mammary glands from mature virgin mice. We observed no change in branch number or ductal extension (Fig. 3B). Next we performed histological analysis on H&E stained mammary gland sections from *MMTV*-*Cre*, *MMTV*-*Cre;Vangl2*^+*/flox*^, and *MMTV*-*Cre;Vangl2*^*flox/flox*^ mice. We found that *MMTV*-*Cre;Vangl2*^*flox/flox*^ glands contained significantly narrower ducts, as measure by luminal width, than *MMTV*-*Cre;Vangl2*^*flox/*+^ or *MMTV*-*Cre* controls (Fig. 3C) (p < 0.03). These results suggest a pronounced defect in duct widening characterized by a near absence of luminal space in the absence of VANGL2.

To determine if changes in cellular size or composition are associated with narrowed ducts, we performed immunostaining using luminal (K8) and basal (K5, SMA) differentiation markers. These analyses found that although *Vangl2*-deficient ducts were narrow, they retained a normal bilayered epithelium consisting of single layers of luminal and basal cells of a similar size and shape to normal control cells (Fig. 3D). The maturation and proper formation of the mammary epithelium is characterized by the specification of distinct apical and basolateral membrane identities^1^. Because VANGL2 is a key cell polarity gene, we next examined the polarization of apical membrane domains and formation of basolateral adherence junctions in these narrow ducts. We observed intact expression of E-cadherin (anti-CDH1) along cell-cell boundaries and apical expression of phosphor-ezrin/radixin/moesin (anti-pERM) along all cell membranes exposed to luminal space (Fig. 3E). Altogether these studies suggest that *Vangl2*-deficient cells are capable of differentiating and responding to key polarization cues during ductal morphogenesis despite abnormal ductal morphogenesis.

We next investigated whether the narrowed ducts were a result of changes in proliferation and/or apoptosis by performing Ki67 and Cleaved Caspase 3 immunohistochemistry on mature virgin *MMTV*-*Cre;Vangl*^*flox/*+^ and *MMTV*-*Cre;Vangl*^*flox/flox*^ glands. We detected significantly lower rates of both proliferation and apoptosis in *Vangl2*-deficient mammary epithelium compared to control (Fig. 3F,G). These findings support previous work suggesting that cellular turnover mediated by mitogenic signaling and pro-apoptotic factors, such as BCL2L11 (BIM), is an essential process for proper lumen formation and maintenance in the mammary gland^20^. During ductal morphogenesis, we also detected lower proliferation within the ducts, but not TEBs, of 6 weeks old mice (Supp. Fig. 2A). Taken together, these results suggest that VANGL2 plays a specific role in mammary duct homeostasis, not in cell differentiation or duct elongation, but specifically in duct maturation and lumen expansion, and the defects observed in the absence of VANGL2 may result from inappropriate cell turnover and proliferation.

### Deregulation of VANGL2 function stunts mammary outgrowth

**Figure 4:**
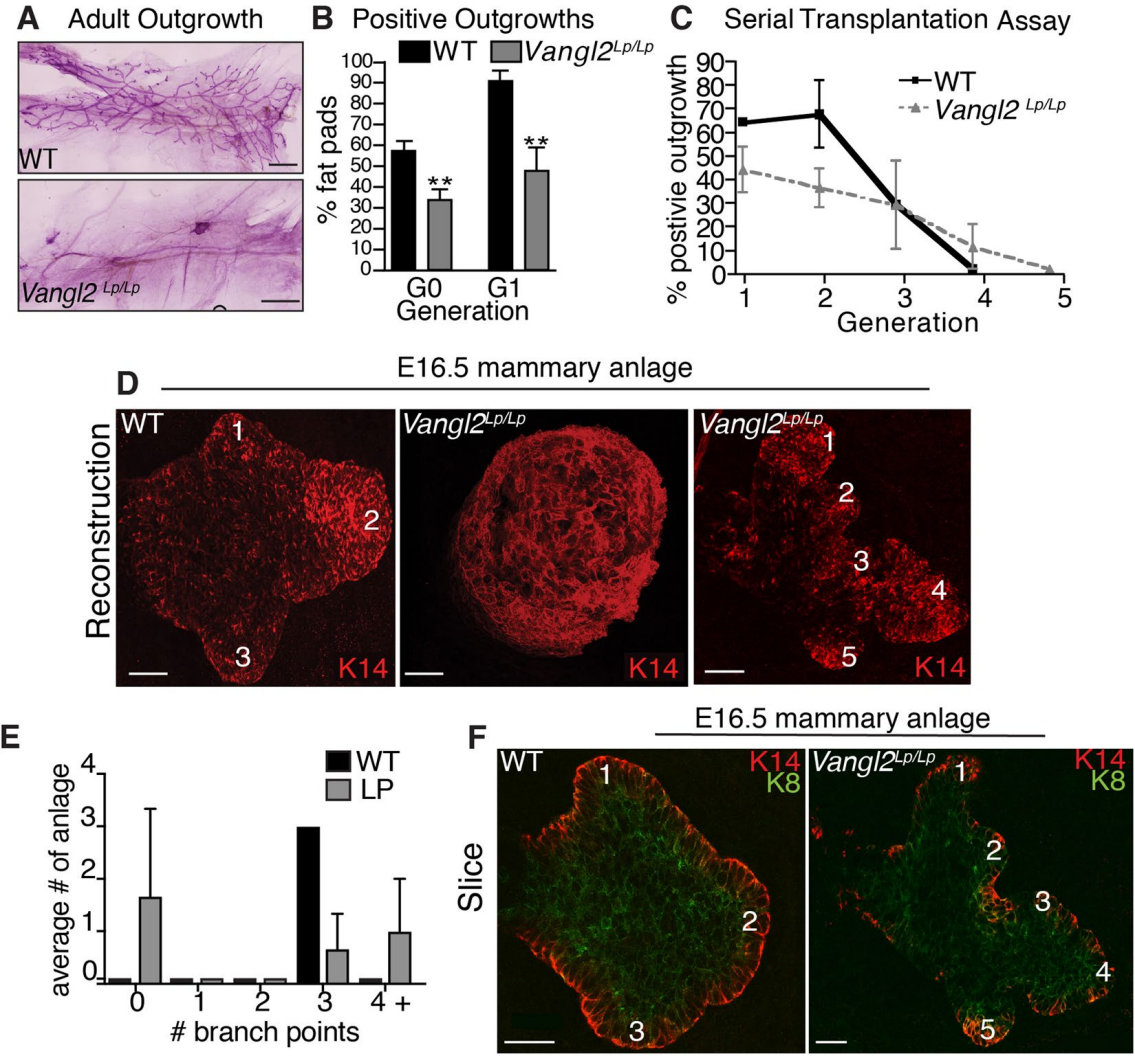
Limited mammary transplant reconstitution capacity and embryonic anlagen defects in *Vangl2*^*Lp/Lp*^ mice. Representative images (**A**) and quantification (**B**) of ductal outgrowth in carmine stained *WT* and or *Vangl2*^*Lp/Lp*^ tissue 12 weeks post-transplantation. For G0 (n = 9 *WT* embryos and 6 *Vangl2*^*Lp/Lp*^ embryos) and G1 (n = 45 mice with contralateral *WT* and *Vangl2*^*Lp/Lp*^ outgrowths). (**C**) Fragments from *WT* and *Vangl2*^*Lp/Lp*^ outgrowths were serially transplanted at 8 week intervals (generations) (n = 5 mice per generation). (**D**) Representative images of *WT* (left) and *Vangl2*^*Lp/Lp*^ (right) E16.5 mammary anlage immunostained with K14 display no branching and hyperbranching phenotypes in the mutant. (**E**) Quantification of sprouting in *WT* and *Vangl2*^*Lp/Lp*^ anlage. (**F**) Representative images of *WT* (left) and *Vangl2*^*Lp/Lp*^ (right) E16.5 mammary anlage immunostained with K14 and K8 show bi-layered structures. Numbers denote branches. n = 8 *WT* and 8 *Vangl2*^*Lp/Lp*^ embryos. Scale bars represent 40 μm. Data are represented as mean ± SEM. Students t-test **p < 0.01.

Next, we investigated disruption of both *Vangl1* and *Vangl2* by examining the well-characterized Looptail (*Vangl2*^*Lp/Lp*^) mice, which harbor a missense mutation in the *Vangl2* gene that interrupts both VANGL1 and VANGL2 trafficking to the cell surface^21,22^. This mutation results in severe PCP defects in multiple organs, leading to embryonic lethality^23^. To study the mammary gland, which develops postnatally, we followed standard protocols to generate *Vangl2*^*Lp/Lp*^ mammary outgrowths via anlage rescue^24^. This entailed harvesting E16.5 mammary anlage from *Vangl2*^*Lp/Lp*^ and *WT* embryos and contralaterally transplanting them into adult hosts that had been pre-cleared of endogenous mammary epithelium. To determine if VANGL1 was altered in the *Vangl2*^*Lp/Lp*^ mammary epithelium, we immunostained sectioned *WT* and *Vangl2*^*Lp/Lp*^ tissue with anti-VANGL1 and found reduced levels of VANGL1, especially along the cell borders in the luminal compartment, in the mutant compared to *WT* (Supp. Fig. 2B). Whole mount analysis of tissue harvested 12-weeks post-transplantation showed that tissue transplanted from *Vangl2*^*Lp/Lp*^ embryos generated fewer outgrowths compared to *WT* (Fig. 4A). To quantify, we defined no outgrowth as fat pads having less than 10% of their area filled with epithelium. Our analysis found that 32% (7/22) of fat pads transplanted with *Vangl2*^*Lp/Lp*^ anlage had outgrowths compared to 60% (18/30) of *WT* (Fig. 4B). To address whether this phenotype was due to transplantation of embryonic tissue, we performed a secondary transplant by harvesting a tissue fragment from each of the 7 mature *Vangl2*^*Lp/Lp*^ and 5 mature *WT* outgrowths and transplanting it into a new host fat pad. We again observed deficient transplantability of *Vangl2*^*Lp/Lp*^ tissue. In this experiment, 44% (20/45) of fat pads containing *Vangl2*^*Lp/Lp*^ transplants generated an outgrowth compared to 93% (42/45) of those transplanted with *WT* (Fig. 4B). These data suggest that the effect of VANGL2 signaling on outgrowth potential persists from embryonic to adult tissue and could be influencing the stem cell population that drives embryonic and postnatal growth.

Given that stem cells, residing in the basal layer, drive outgrowth in the mammary gland and that our results show expression of VANGL2 in basal cells and stunted growth in *Vangl2*^*Lp/Lp*^ outgrowths, we investigated the role of VANGL2 in stem cell self-renewal activity. Adult stem cells in the mammary gland are responsible for fueling postnatal growth of the gland. Previous studies have shown that mammary stem cell self-renewal can be assayed by serial transplantation whereby stem cells are challenged with growing an entire gland after each passage of a tissue fragment^25,26^. To this end, we serially transplanted contralateral *WT* and *Vangl2*^*Lp/Lp*^ outgrowths and found they lost regenerative capacity at generation 4 and 5, respectively, suggesting that VANGL2 does not significantly alter tissue passageability. However, consistent with our previous data, *WT* tissue transplants generated 1.5 times more positive outgrowths in the first two passages compared to *Vangl2*^*Lp/Lp*^ (Fig. 4C). Taken together, these data suggest that loss of cell surface VANGL proteins significantly reduces the outgrowth potential, but not the self-renewal capacity, of stem cells.

### *Vangl2*^*Lp/Lp*^ embryos contain either stunted anlage or anlage containing supernumerary embryonic sprouts

The large percentage of *Vangl2*^*Lp/Lp*^ anlage rescue experiments that did not produce mammary outgrowths suggests that development of the mammary anlage may be compromised in *Vangl2*^*Lp/Lp*^ animals. To investigate, we examined *Vangl2*^*Lp/Lp*^ mammary rudiments in intact embryos. By E16.5 the mammary gland is specified and has undergone bifurcation to generate 3 sprouts^4^. Using fluorescent whole mount immunostaining with anti-K14, we visualized the overall structure of E16.5 *WT* and *Vangl2*^*Lp/Lp*^ mammary anlage. Three-dimensional reconstruction revealed 100% of *WT* anlage produced three sprouts (Fig. 4D,E), whereas 50% of *Vangl2*^*Lp/Lp*^ anlage had no sprouts, generating instead a large bud-like structure (Fig. 4D,E). This observation suggests that the lack of VANGL on the cell surface compromises the ability of the embryonic mammary bud to break symmetry, bifurcate and generate branches. Furthermore, the lack of prenatal branching in 50% of examined embryo anlage likely relates to the remarkably similar incomplete penetrance we observed in the outgrowth efficiency of transplanted *Vangl2*^*Lp/Lp*^ anlage (Fig. 4A,B).

Next, we examined the 50% of *Vangl2*^*Lp/Lp*^ anlage that developed past the tissue bud stage and formed branched structures. We found that only 40% of these *Vangl2*^*Lp/Lp*^ anlage formed a typical rudimentary tree with 3 distinguishable sprouts, similar to the 3-pronged structure of WT anlage (Fig. 4E). The remaining 60% of *Vangl2*^*Lp/Lp*^ anlage contained 4 or more sprouts (Fig. 4D,E). Whole mount immunohistochemistry with basal (anti-K14) and luminal (anti-K8) cell markers revealed that both *WT* and *Vangl2*^*Lp/Lp*^ anlage are composed of K8 and K14 positive cells, suggesting bilayer formation was unaffected by loss of surface VANGL function (Fig. 4F). Together, the data demonstrate a role for VANGL signaling in generating proper tissue structure during the critical early stages of mammary gland development.

### *Vangl2*^*Lp/Lp*^ outgrowths display supernumerary end buds and ectopic acini

**Figure 5:**
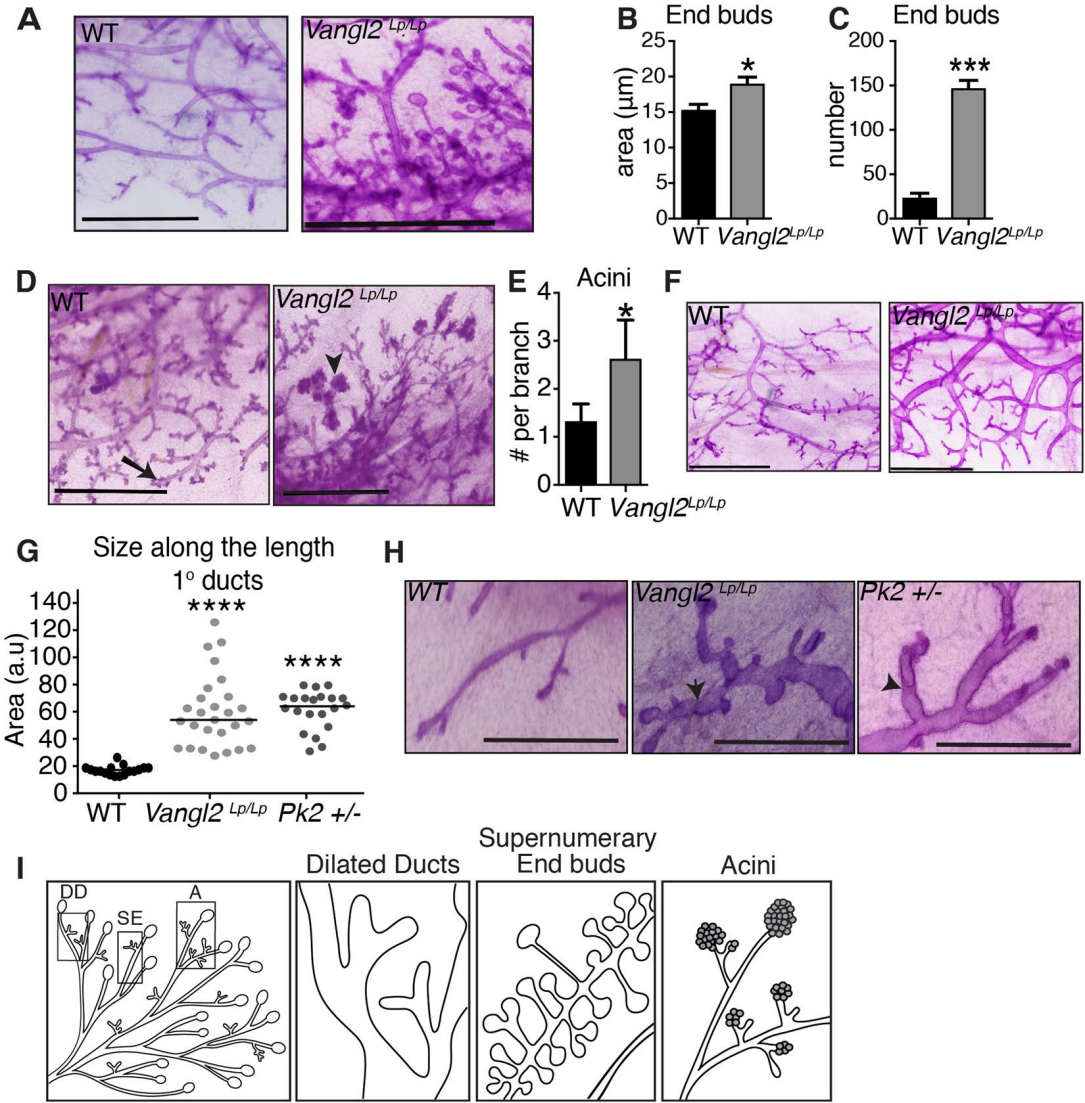
Vangl2^Lp/Lp^ outgrowths display distinct morphological defects. (**A**) Magnified view of supernumerary end buds in *Vangl2*^*Lp/Lp*^ tissue from carmine stained, contralaterally transplanted *WT* and *Vangl2*^*Lp/Lp*^ outgrowths 12 weeks post-transplantation; *WT* outgrowth contains no supernumerary end buds. Quantification of the area (**B**) and number (**C**) of end buds in *WT* and *Vangl2*^*Lp/Lp*^ outgrowths. (**D**) Magnified view of acini in *Vangl2*^*Lp/Lp*^ tissue from carmine stained, contralaterally transplanted *Vangl2*^*Lp/Lp*^ tissue; *WT* outgrowth contains no acini. Arrow shows tertiary branching. Arrowhead denotes acinar structure. (**E**) Quantification of the number of acini in *WT* and *Vangl2*^*Lp/Lp*^ outgrowths. (**F**) Magnified view of dilated ducts in tissue from carmine stained, contralaterally transplanted *WT* and *Vangl2*^*Lp/Lp*^ outgrowths, harvested at 12 weeks; *WT* outgrowth shows smooth ductal borders with no constrictions. (**G**) Quantification of ductal size in *WT*, *Vangl2*^*Lp/Lp*^ *and Pk2* +/− tissue (n = 10 ducts from 3 mice per genotype). (H) Representative images of carmine stained *WT* outgrowth, *Vangl2*^*Lp/Lp*^ outgrowth and *Pk2* +/−  ducts and displaying pinched ductal phenotype. Arrows denote constrictions. (I) Cartoon representation of the distinct phenotypes observed in *Vangl2*^*Lp/Lp*^ outgrowths: DD, dilated ducts; SE, supernumerary end buds; A, acini. Scale bars represent 1.5 mm. Data are represented as mean ± SEM. Students t-test *p < 0.05, ***p < 0.001, and ****p < 0.0001.

Given that TEBs are the site of growth and proliferation in the mammary gland and VANGL2 is highly expressed in this region, we performed additional transplantation experiments to evaluate the postnatal consequences of VANGL loss-of-function in successful mammary outgrowths. We observed three distinct phenotypes in *Vangl2*^*Lp/Lp*^ outgrowths (n = 20 *WT/LP* pairs) that were non-mutually exclusive. One phenotype was an abundance of end buds. We found that 45% (9/20) of *Vangl2*^*Lp/Lp*^ outgrowths contained 6-fold more TEBs that were significantly larger compared to *WT* (Fig. 5A–C,I, Supp. Fig. 2C). Together, these findings suggest that VANGL2 is expressed in a gradient across the gland and loss of this graded expression enhances TEB formation.

A second phenotype (8/20) was the formation of premature acini (Fig. 5D,E,I). Our transplantation assays were performed such that *WT* fragments were contralaterally transplanted into the same mouse as *Vangl2*^*Lp/Lp*^ tissue. Therefore, both tissues were exposed to the same hormonal environment so this defect does not represent cyclic side branching. Nevertheless, we found that *Vangl2*^*Lp/Lp*^ outgrowths contained twice as many acini per duct, compared to *WT* (Fig. 5D,E,I, Supp. Fig. 2D). To evaluate the morphology of these structures, we sectioned *WT* and *Vangl2*^*Lp/Lp*^ tissue and immunostained with anti-CDH1, revealing a single layer of luminal cells surrounding a hollow lumen (Supp. Fig. 2E). In contrast, multiple layers of disorganized luminal cells frequently occluded *Vangl2*^*Lp/Lp*^ acini (Supp. Fig. 2E). Thus, *WT* acini morphologically mimicked normal tertiary branching, whereas *Vangl2*^*Lp/Lp*^ acini showed exuberant, disorganized, tertiary acini, despite being exposed to the same hormonal environment.

A third phenotype (6/20) was the presence of dilated ducts (Fig. 5G–I, Supp. Fig. 2F). We quantified the size of these ducts along their length by optically dividing each duct into horizontal sections using a grid and found that the average area of *Vangl2*^*Lp/Lp*^ ducts was 3 times greater than *WT* (Fig. 5G). In addition to their increased size, *Vangl2*^*Lp/Lp*^ ducts are also marked by pronounced constrictions along their lengths, giving the ducts a pinched appearance (Fig. 5H). In contrast, *WT* ducts are uniform in size; even if their area is expanding or contracting, the change is gradual (Fig. 5H). This difference was reflected in the quantification where we observed *WT* measurements clustering around 20 a.u., whereas *Vangl2*^*Lp/Lp*^ measurements were more variable, echoing the abrupt changes in ductal size occurring over the length of *Vangl2*^*Lp/Lp*^ ducts (Fig. 5G,H). Actin polymerization plays an important role in regulating lumen formation, and disorganized actin patterns have been observed in tubular and ductal organs of *Vangl2*^*Lp/Lp*^ animals^7–9^. To evaluate the filamentous actin cytoskeleton, we sectioned and phalloidin stained WT and *Vangl2*^*Lp/Lp*^ tissue, observing irregular f-actin organization and reduced staining, particularly in the luminal compartment of *Vangl2*^*Lp/Lp*^ ducts (Supp. Fig. 2G). In order to investigate whether this phenotype was due to a loss in PCP signaling, we examined the morphology of mammary glands from another PCP mutant mouse, *Prickle2* (*Pk2*). PK2 is a cytoplasmic mediator of PCP signaling downstream of VANGL2^27^. Immunostaining of sectioned glands obtained from mature virgin mice with anti-PK2 antibodies revealed cytoplasmic and membrane staining in a pattern similar to VANGL2 expression (Supp. Fig. 2H). *Pk2* mutant mice have an embryonic lethal phenotype, resulting in termination around E3.5 before the mammary rudiment is formed^28^. This prevented anlage rescue; therefore instead, we harvested mammary glands from adult virgin *WT* and *Pk2*^+/−^ mice and performed whole mount analysis. Similar to *WT*, we found that *Pk2*^+/−^ glands filled 100% of the fat pad (Supp. Fig. 2I), but *Pk2*^+/−^ ducts were dilated except for constrictions, a phenotype also observed in *Vangl2*^*Lp/Lp*^ outgrowths (Fig. 5G,H). Taken together these results suggest that loss of surface VANGL signaling regulates mammary ductal diameter.

### Depletion of VANGL2 in basal versus luminal cell populations alters cyst formation

**Figure 6:**
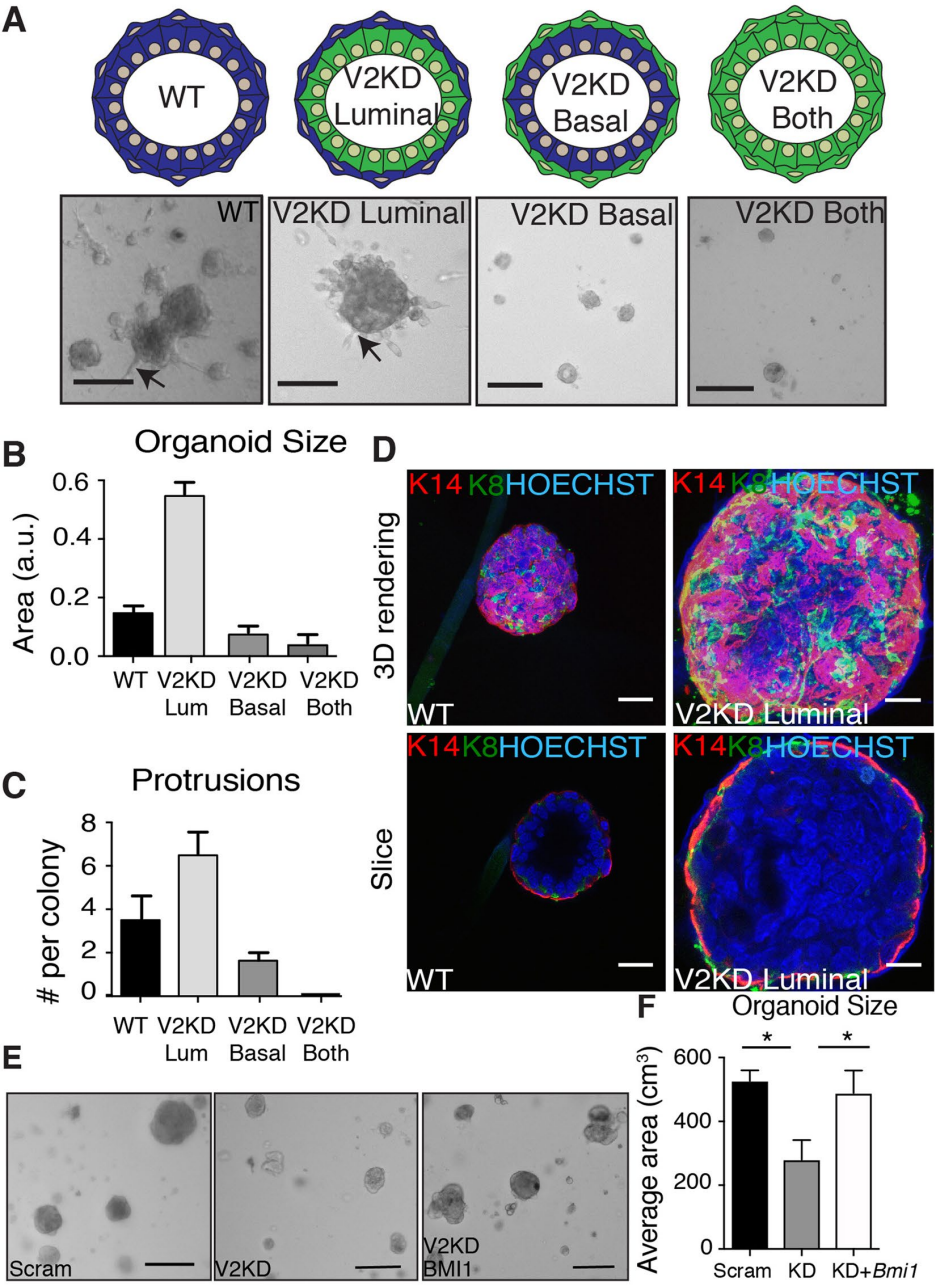
Knockdown of *Vangl2* in the basal versus luminal layers of cysts alter morphology. (**A**) Schematic (top) and representative images (bottom) of mosaic cysts after 4 days in culture. Quantification of cyst size (**B**) and number of protrusions (**C**) in the mosaic cysts. (**D**) Immunostaining of K14 (red) and K8 (green) showing the surface (top) or inside (bottom) of *WT* or mosaic cysts with *Vangl2* knocked down in only luminal cells. Representative images (**E**) and quantification (**F**) of rescue of *Vangl2* KD cyst size with overexpression of *Bmi1*. n = 3 biological replicates. Scale bars represent 20 μm. Data are represented as mean ± SEM. Students t-test *p < 0.05.

Expression of VANGL2 in both the luminal and basal subpopulations, and the range of phenotypes displayed in the *Vangl2*^*Lp/Lp*^ outgrowths, suggest that VANGL2 is governing different aspects of mammary gland development, perhaps by differentially influencing cell interactions and signaling in the distinct basal and luminal compartments of the mammary gland. To test this hypothesis, we used differential trypsinization to separate the two subpopulations^29^ and lentiviral-mediated knock down to reduce expression of *Vangl2* in either the basal, luminal or both cell types (Supp. Fig. 3A,B,4). By mixing knock down (KD) and *WT* subpopulations of cells and culturing them in Matrigel in the absence of growth factors, we generated mammary cysts that were mosaic in the expression of VANGL2, such that either the basal or luminal compartment was VANGL2-deficient (Fig. 6A). As a control, we also generated KD and *WT* cysts in which both or neither compartments were VANGL2-deficient (Fig. 6A). *WT* cells formed round cysts with approximately 3 protrusions, whereas KD cysts were half the size with no protrusions (Fig. 6B,C). KD of *Vangl2* in the luminal layer produced cysts similar in overall appearance to *WT* but 3-fold larger (Fig. 6A,B). Consistent with this increase in size, the KD cysts had 2-fold more cell protrusions (Fig. 6A,C). We immunostained these cysts with lineage markers K14 and K8 (Fig. 6D). Optical sections through these cysts show that, while *WT* cysts have clear lumens, *Vangl2* luminal KD cysts contained a filled lumen. These data show that loss of VANGL2 in the luminal population leads to disorganization, which echoes the TEB and acinar phenotypes in the *Vangl2*^*Lp/Lp*^ full outgrowths and the narrowed lumen phenotype in the *MMTV*-*Cre;Vangl2*^*flox/flox*^ glands. In contrast, KD of *Vangl2* in the basal layer, which contains a subpopulation of mammary stem cells, yielded cysts that resembled the full *Vangl2* KD cysts: half the size of *WT* with half the number of protrusions (Fig. 6A–C). The small size and number of these basal cysts prevented us from successfully immunostaining them. These studies suggest that VANGL2 has different functions in the luminal and basal compartments, restricting cyst formation in the luminal compartment while promoting cyst formation in the basal compartment.

To further understand VANGL2 signaling in the mammary gland, we performed RT-qPCR on cDNA generated from *Vangl2*^*Lp/Lp*^ and *WT* outgrowths and mammary tissue derived from *MMTV*-*Cre;Vangl2*^*flox/flox*^ and *MMTV*-*Cre* only mice to evaluate the expression of genes encoding proteins that mediate the downstream signaling functions of VANGL2. To probe canonical WNT signaling, we measured expression of *Axin2*. In addition, as previous studies have shown that VANGL2 is a regulator of SHH, we evaluated SHH signaling by measuring the expression of downstream effectors *Bmi1* and *Ptch1*^30^. Although we found no change in *Axin2* or *Ptch1* expression in *Vangl2*^*Lp/Lp*^ outgrowths compared to *WT* (Supp. Fig. 3C), we did observe a decrease in both *Axin2* and *Ptch1 in MMTV*-*Cre;Vangl2*^*flox/flox*^ compared to *MMTV*-*Cre* only mice (Supp. Fig. 3D). Importantly we observed a significant and consistent down regulation of *Bmi1* in both *Vangl2*^*Lp/Lp*^ outgrowths and *MMTV*-*Cre;Vangl2*^*flox/flox*^ mammary tissue compared to respective *WT* controls (Supp. Fig. 3C,D). BMI1 is part of the polycomb repressive complex 1 (PRC1) that maintains genes in a transcriptional repressive, quiescent state. Loss of its expression in *Vangl2*^*Lp/Lp*^ tissue could explain our difficulty rescuing *Vangl2*^*Lp/Lp*^ anlage, propagating the tissue *in vivo* and generating cysts with KD of *Vangl2* in the basal, stem cell containing compartment of the mammary gland. To investigate, we overexpressed *Bmi1* in *Vangl2* KD cysts and found that reconstitution of *Bmi1* expression rescued the formation of cysts that were the same size as those generated with scrambled shRNA (Fig. 6E,F). These results show an inverse relationship between VANGL2 and *Bmi1* expression and suggest this may regulate the viability and outgrowth of mammary cells.

## Discussion

Although non-canonical WNT signaling has been shown to have an important role in normal mammary gland development, the mechanisms underlying the process remain elusive^31^. WNTs have both diverse and vast influences on mammary morphogenesis that can be explained by their ability to engage various receptors. In this study, we address non-canonical WNT/PCP signaling by directly examining the consequences of aberrant VANGL receptor function. We found that both VANGL1 and VANGL2 are expressed in the mammary gland and can be detected by immunostaining in the same cell populations; however, their loss-of-function phenotypes are different. We discovered that VANGL2 plays a more important role in both embryonic and postnatal gland development compared to VANGL1. We also observed distinct VANGL2 phenotypes depending on the genetic approach we used. Conditional loss of VANGL2 during puberty in the *MMTV*-*Cre; Vangl2*^*flox/flox*^ mice led to defects in post-natal mammary duct morphogenesis whereas aberrant VANGL2 signaling during embryonic development in *Vangl2*^*Lp/Lp*^ transplants resulted in a range of embryonic and differentiation defects consistent with impaired stem cell function.

One phenotype shared between both of our models is mis-regulation of ductal size. The non-canonical Wnt/PCP pathway regulates ductal diameter in a variety of epithelia by controlling two morphogenetic processes: convergent extension (CE) and oriented cell divisions (OCD). In this way, length-wise extension of tubules through OCDs is balanced with the reorganization of cells along the width via CE, and, together, these mechanisms regulate ductal diameter. The *MMTV*-*Cre;Vangl2*^*flox/flox*^ mammary glands display narrow ducts, whereas *Vangl2*^*Lp/Lp*^ outgrowths contain ducts that are either uniformly wide or wide but marked with constrictions. These phenotypes suggest a role for VANGL2 in regulating mammary ductal diameter and are consistent with luminal width defects previously observed in *Vangl2* mutant mice. For example, embryonic and adult airway lumen in the lungs of *Vangl2*^*Lp/Lp*^ and *Vangl2*^*Lp/*+^ mice, respectively, are absent or narrow^8,32^, a phenotype that is reminiscent of the narrow lumens observed in *MMTV*-*Cre;Vangl2*^*flox/flox*^ mammary glands (Fig. 3C). In contrast, dilated lumens are observed in both Podocin-Cre;*Vangl2*^*flox/flox*^ and *Vangl2*^*Lp/Lp*^ embryonic kidneys^7,9,10^. Recent studies show that OCDs are significantly off axis in *Vangl2*^*Lp/Lp*^ embryonic kidneys compared to *WT*, leading to wider lumens^11^. Thirty percent of *Vangl2*^*Lp/Lp*^ mammary glands display dilated ducts, with some containing points of marked narrowing (Fig. 5F–H). Thus, it is likely that VANGL2 controls OCDs during mammary morphogenesis, but confirmation will require analysis of cell divisions and movements that occur over a longer time frame in the postnatal mammary gland. In addition, a number of other mechanisms have been implicated in mammalian lumen size control including RhoGTPase regulated actomyosin contractility, a known downstream effector of PCP signaling activity, and fluid-driven lumen expansion^33^. We did observe reduced proliferation in *MMTV*-*Cre;Vangl2*^*flox/flox*^ mammary glands (Fig. 3F), which may contribute to abnormal duct formation in the context of aberrant OCDs. In support of these findings *in vivo*, a similar reduction in proliferation with *Vangl2* depletion was previously reported in two basal breast cancer lines^3^, and we observed that *Vangl2* deficiency, specifically in basal cells, impairs the growth of the entire cyst (Fig. 6A–C).

The fact that we observed different ductal diameter phenotypes in the *MMTV*-*Cre;Vangl2*^*flox/flox*^ mammary gland versus *Vangl2*^*Lp/Lp*^ outgrowths may be due to the timing of VANGL2 inactivation in these different models, the nature of the mutation, or both. Conditional loss of *Vangl2* was achieved using a Cre driver activated during puberty and, while it resulted in a major reduction in VANGL2 protein^18^, this decrease occurred after birth. In contrast, the endogenous missense mutation in the Looptail version of VANGL2 influences both embryonic and postnatal development. In the embryonic neuroepithelium and regenerating adult muscle, VANGL2 governs the asymmetric cell division of stem/progenitor cells by OCD^34,35^. In the mammary gland, VANGL2 is expressed in the stem cell-containing, basal cell population. Disruption of mammary stem cell self-renewal, expansion and/or cell fate acquisition during embryogenesis could alter the cellular dynamics of the developing gland and dramatically influence ductal morphogenesis in ways that are distinct from the loss of VANGL2 during puberty. Moreover, recent findings describe VANGL proteins functioning together in a heteromeric protein complex that prevents trafficking of VANGL1 to the cell surface in *Vangl2*^*Lp/Lp*^ mice^22,36^. Indeed, we observed such disruption of VANGL1 localization at the cell borders of *Vangl2*^*Lp/Lp*^ tissue (Supp. Fig. 2B) This dual disruption can result in mislocalization of other PCP components such as Pk2, and lead to more dominant phenotypic traits^22^. Thus, the cell surface loss of both VANGL2 and VANGL1 from conception is likely to adversely affect both CE and OCD, and may explain the 50% decline in outgrowth potential, and the dilated ducts and other morphological phenotypes displayed in the successful *Vangl2*^*Lp/Lp*^ outgrowths compared to *MMTV*-*Cre;Vangl2*^*flox/flox*^ mammary glands.

A surprising result from this study was the identification of a link between the expression of BMI1 and VANGL2. We show that loss of VANGL2 reduces *Bmi1* expression and that overexpression of *Bmi1* rescues mammary cyst formation. BMI1 is involved in the self-renewal and maintenance of stem cells^37,38^, and BMI1 KO mice have mammary glands that are stunted similar to *Vangl2*^*Lp/Lp*^ outgrowths^38^. Furthermore, the stem cell-containing, basal cell population of the mammary gland expresses VANGL2 and previous studies have shown that VANGL2 is needed for symmetric expansion of stem cell division^34,35^. Because the Looptail (*Vangl2*^*Lp/Lp*^) mutation is lethal, we performed anlage rescue, and this gave us our first indication of a potential stem cell defect in *Vangl2*^*Lp/Lp*^ tissue. Compared to WT, there was a 50% reduced rescue of *Vangl2*^*Lp/Lp*^ anlage, and a similar reduction in tissue viability with subsequent fragment passage. We also observed this decreased viability in our cyst assay when we reduced the level of *Vangl2* expression in basal cells. We performed an *in vivo* serial transplantation assay that did not, however, reveal a difference in the passageability of WT and *Vangl2*^*Lp/Lp*^ tissue. Although this result suggests that stem cells are not adversely affected by the loss of cell surface VANGL in *Vangl2*^*Lp/Lp*^ tissue, an alternate explanation is that rescued tissue contains sufficient functional VANGL2 to preserve, at least some, mammary stem cell viability. Dosage sensitivity of the *Vangl2*^*Lp*^ allele is well documented^39^, and supported in this study by the phenotypic heterogeneity observed in the mammary outgrowths produced by rescued anlage. Thus, it is tempting to speculate that VANGL2 may promote the expansion and/or self-renewal of mammary stem cells in the embryo and loss of VANGL2 signaling results in the depletion of the stem cell pool. Current experiments are aimed at understanding if VANGL2 is regulating stem cell number in the mammary gland using the null allele. Furthermore, it will be interesting to determine whether such deregulation of mammary stem cell self-renewal, expansion and/or cell fate acquisition by VANGL2 may contribute to its cancer modulatory effects.

## Methods

### Mouse strains

*Vangl2*^*Lp/Lp*^ mice were obtained from Jackson Laboratory. *Vangl1CKO* and *Pk2*+*/−* mice were generated as previously described^15,28^. *Vangl2*^*flox/flox*^ mice were generated as previously described^18^ and were crossed with Tg(MMTV-cre)1Mam (line A) provided by K Wagner^19^. This research was both approved by and conducted in accordance with the guidelines set by the University of California, Santa Cruz animal care committee (IACUC) and the Peter MacCallum Cancer Centre Animal Experimental Ethics. The research was conducted in compliance with National Health and Medical Research Council (Australia) guidelines.

### Mammary gland transplantation

Mammary anlage were dissected from *Vangl2* ^Lp/Lp^ embryos, and transplanted into pre-cleared fat pads of *Foxn1*^*nu*^ mice^40^. Contralateral outgrowths were harvested 12 weeks post-transplant and subjected to whole mount carmine staining. For serial studies, epithelial fragments were harvested and transplanted into a new host every 12 weeks.

### *In vivo* branch quantification

Primary branches were defined as ducts extending from the site of transplantation and terminating in an end bud. Secondary and tertiary branches were defined as branches extending from primary ducts or secondary branches, respectively. Branch number was quantified by tracing the primary ductal structure and counting the number of primary ducts and secondary/tertiary branches in Fiji^41^.

### Ductal size quantification

To quantify the size of ducts in wholemounts, we measured the area of each duct in whole mount images using Fiji. A grid comprised of vertical lines spaced equidistant was laid on top of whole mount images. This provided a way to divide the ducts into equal sections. Next, single ducts were outlined and the space (area) occupied by that duct in each section was quantified. To quantify duct width at the histological level, all H&E stained duct cross-sections (24–51 ducts/mouse, 3 mice/group), were captured on a BX51-P Olympus Microscope and the maximum width of each duct measured using Metamorph Image Analysis software (Molecular Devices).

### Immunohistochemistry and Immunofluorescence

IHC was performed as previously described with: Hoechst (AnaSpec, #AS-83218), anti-E-cad (Cell Signaling, #3195 S or BD biosciences, 610182), anti-VANGL2 (SCBT, #46561), anti-VANGL1 (R&D, #HPA02235), Ki67 (Novus, NB500-170), Cleaved Caspase 3 and pERM (Cell Signaling #9661, #3726), Phalloidin (Invitrogen, #A12381), aSMA (DAKO, #M0851), anti-K8 (Developmental Studies Hybridoma Bank, Troma-1), Cytokeratin 5 (Covance, #PRB-160P), and anti-K14 (Covance, #PRB-155P). Images were collected on a Leica SP5 confocal microscope. Brightfield imaging was performed on a Biorevo BZ-9000 Digital Microscope (Keyence) and confocal microscopy performed on a Nikon C2 Confocal, Leica SP5 confocal and Solamere Spinning Disk Confocal Microscope. 3-dimensional reconstructions were performed using Fiji or NIS-elements.

### RNA extraction and RT-qPCR

Total RNA was isolated from MEC and LEC primary cell fractions (separated as described^29^) or from FACS-purified basal (Lin**_**CD24 + CD29-high) and luminal (Lin**_**CD24 + CD29low) cells using TRIzol reagent (Invitrogen) and prepared as previously described^29^. cDNA was prepared from 1 μg RNA using an iScript cDNA synthesis kit (Bio-Rad). RT-qPCR was performed in triplicate using LightCycler 480 SYBR Green I Master (Roche) and quantified using either Rotor Gene 6000 real-time PCR machine and software or Bio-Rad CFX Connect Real-Time System and CFX Manager software (Bio-Rad). Samples were normalised to GAPDH and expression levels calculated using the 2−ΔCt method. Real-time primers listed in S1 Table. Results were normalized to that of *Gapdh*.

### Transduction of primary mammary epithelial cells

Luminal and myoepithelial primary MECs were isolated by differential trypsinization as previously described^29^. Next, transduction of primary MECs was performed as described previously^42^. Single MECs were seeded at a density of 500,000 cells in a 24-well low-attachment plate and infected at an MOI of 30 with designated lentivirus in 800 µl of growth media. Cells were infected overnight for ∼16 h and washed three times with PBS and re-plated.

### Primary mouse mammary epithelial cell cyst assay

3D cultures were generated using the “on top” Matrigel method as previously described^43^. Briefly, transduced luminal and myoepithelial cells were trypsinized into a single cells suspension. The cells were then mixed in a 1:4 myoepithelial: luminal ratio and seeded at a final density of 25,000 cells on Matrigel-coated 8 well chamber slides. Cells were allowed to attach to the surface of the Matrigel for 30 mins at 37 °C. A final volume of 10% Matrigel in growth media was added to the cells after attachment. Cells were imaged and analyzed for cyst formation after 72 hrs.

### Western blot analysis

Cell or whole tissue lysates were prepared and analyzed by western blot as previously described. Luminescence was captured onto x-ray film or by the Biorad Gel Doc XR+ imaging system. Images were then quantified using Image Lab with band densitometry normalized to GAPDH.

### Statistical analysis

Statistical analyses (Student’s t-test and ANOVA) were conducted on Graphpad Prism. Significance is indicated as *p < 0.05, **p < 0.01, ***p < 0.001, ****p < 0.0001. Bars represent SEM.

## Supplementary information

Supplementary Information

## Data Availability

The datasets generated during and/or analysed during the current study are available in the Gene Expression Omnibus (GEO) Reference ID: GSE19446.
