# Supplementary material for: VANGL2 regulates luminal epithelial organization and cell turnover in the mammary gland
Source: Sci Rep. 2019 May 8;9:7079. doi: 10.1038/s41598-019-43444-8 (PMC6506599; doi:10.1038/s41598-019-43444-8)
Supplement: Supplementary file 1 — Supplementary Information [file 41598_2019_43444_MOESM1_ESM.pdf]

VANGL2 regulates luminal epithelial organization and cell turnover in the mammary gland

Prestina Smith, Nathan Godde, Stefany Rubio, Melawit Tekeste
Eszter K. Vladar, Jeffrey D. Axelrod, Deborah J. Henderson, Michal Milgrom-Hoffman,
Patrick O. Humbert, Lindsay Hinck

SUPPLEMENTAL FIGURE LEGENDS:

Supplemental Figure1: *Vangl1* and *Vangl2* expression in FACS purified mammary cell populations. (A) Analysis of *Vangl1* and *Vangl2* expression within separate FACS purified mammary cell populations using a previously published microarray dataset (GSE19446) and normalised to relative stroma expression. Expression of differentiation markers *SMA* and *K18* confirmed purity of basal and luminal cell populations, respectively.

Supplemental Figure2: VANGL loss of function phenotypes in the mammary gland phenocopy PCP defects. (A) Immunostaining in 6 weeks old *MMTV-Cre;Vangl2*$^{+/+}$ and *MMTV-Cre;Vangl2*$^{flox/flox}$ glands and quantitation of Ki67 or Cleaved Caspase 3 in ducts and TEBs (n=3-5 mice per genotype). Data are represented as mean +/- SEM. Scale bar represents 100µm. Student's t-test *$p<0.05$. (B) Immunostaining of WT and *Vangl2*$^{Lp/Lp}$ tissue showing VANGL1 (green) localization in mammary ducts. (C-D) Representative whole mount images of contralaterally transplanted, carmine stained *WT* and *Vangl2*$^{Lp/Lp}$ outgrowths 12 weeks post-transplantation, showing supernumerary end buds (C) and acini (D). (E) CDH1 immunostaining (red) of *WT* and *Vangl2*$^{Lp/Lp}$ tissue showing altered morphology in *Vangl2*$^{Lp/Lp}$ ducts. Representative whole mount images of contralaterally transplanted, carmine stained *WT* and *Vangl2*$^{Lp/Lp}$ outgrowths 12 weeks post-transplantation, showing dilated ducts (F). (G) Phalloidin (red) staining in *WT* and *Vangl2*$^{Lp/Lp}$ ducts. (H) Immunostaining of *WT* 8-week tissue showing PK2 cytoplasmic and membrane localization in mammary ducts. (I) Representative whole mount images of carmine stained *Pk2*$^{+/-}$ outgrowths 12 weeks post-transplantation display dilated ducts compared to *WT* control. Lu denoted lumen. Scale bars represent 1.5mm (C, D, F, I) and 20µm (B, E, G, H).

Supplemental Figure 3: Depletion of VANGL2 in basal versus luminal cell populations reduces Bmi1 expression. (A) RT-qPCR analysis of *Vangl2* mRNA levels in WT and *Vangl2* knockdown primary cells. (B) Representative immunoblots of VANGL2 and GAPDH (control) in primary cells transduced with two constructs (C1, C2) to knockdown *Vangl2* (n=3). (C) qRT-PCR analysis of *Axin2*, *Ptch2* and *Bmi1* expression in WT and $\mathit{Vangl2}^{Lp/Lp}$ outgrowths. (D) qRT-PCR analysis of *Axin2, Ptch2* and *Bmi1* expression in $\mathit{MMTV\text{-}Cre;Vangl}^{flox/flox}$ mammary glands compared to Cre only control. n=3 biological replicates. Data are represented as mean ± SEM.

Supplemental Figure 4: Full scans of immunoblots used in Figure 1E and Supplemental Figure 3B. Red rectangles delineate regions used in main figures.

Supplemental Table 1: qPCR primers used in these studies.

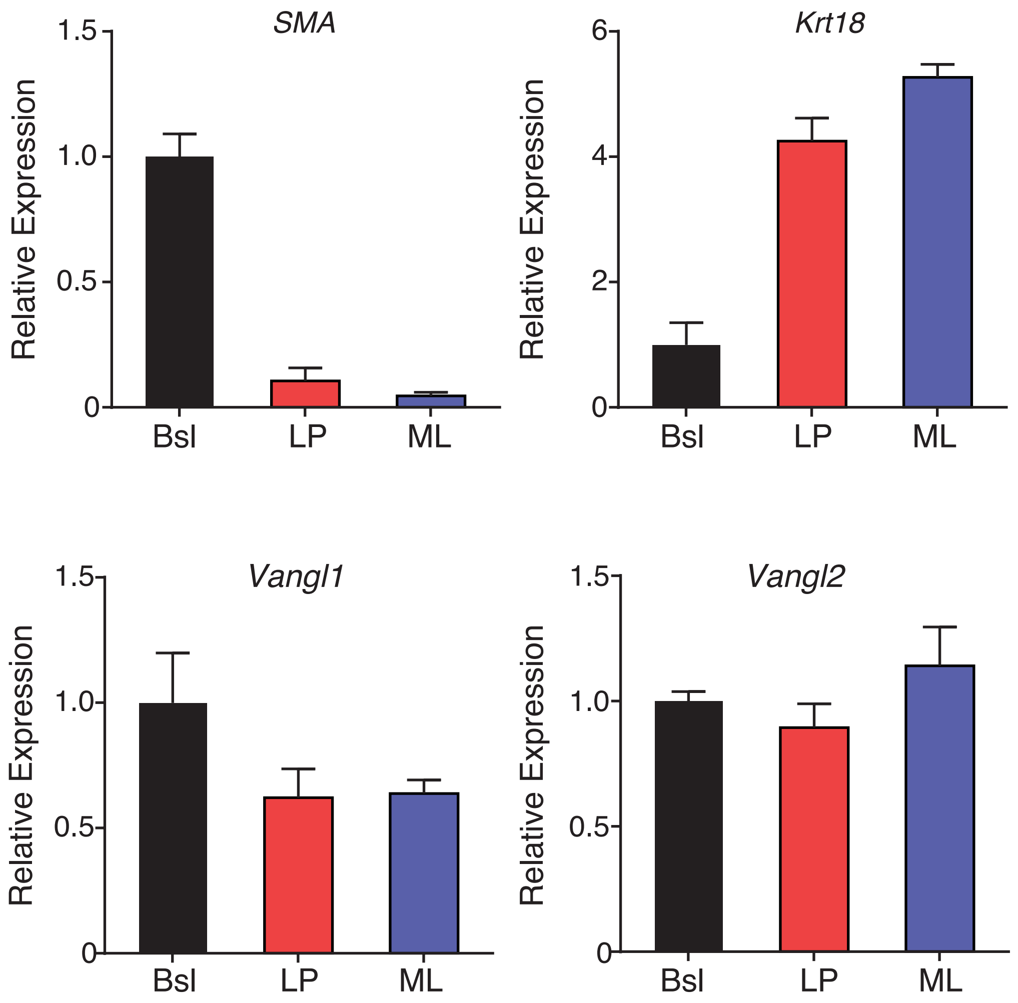
SMA
Krt18
Vangl1
Vangl2
Relative Expression
1.5
1.0
0.5
0
6
4
2
Bsl
LP
ML

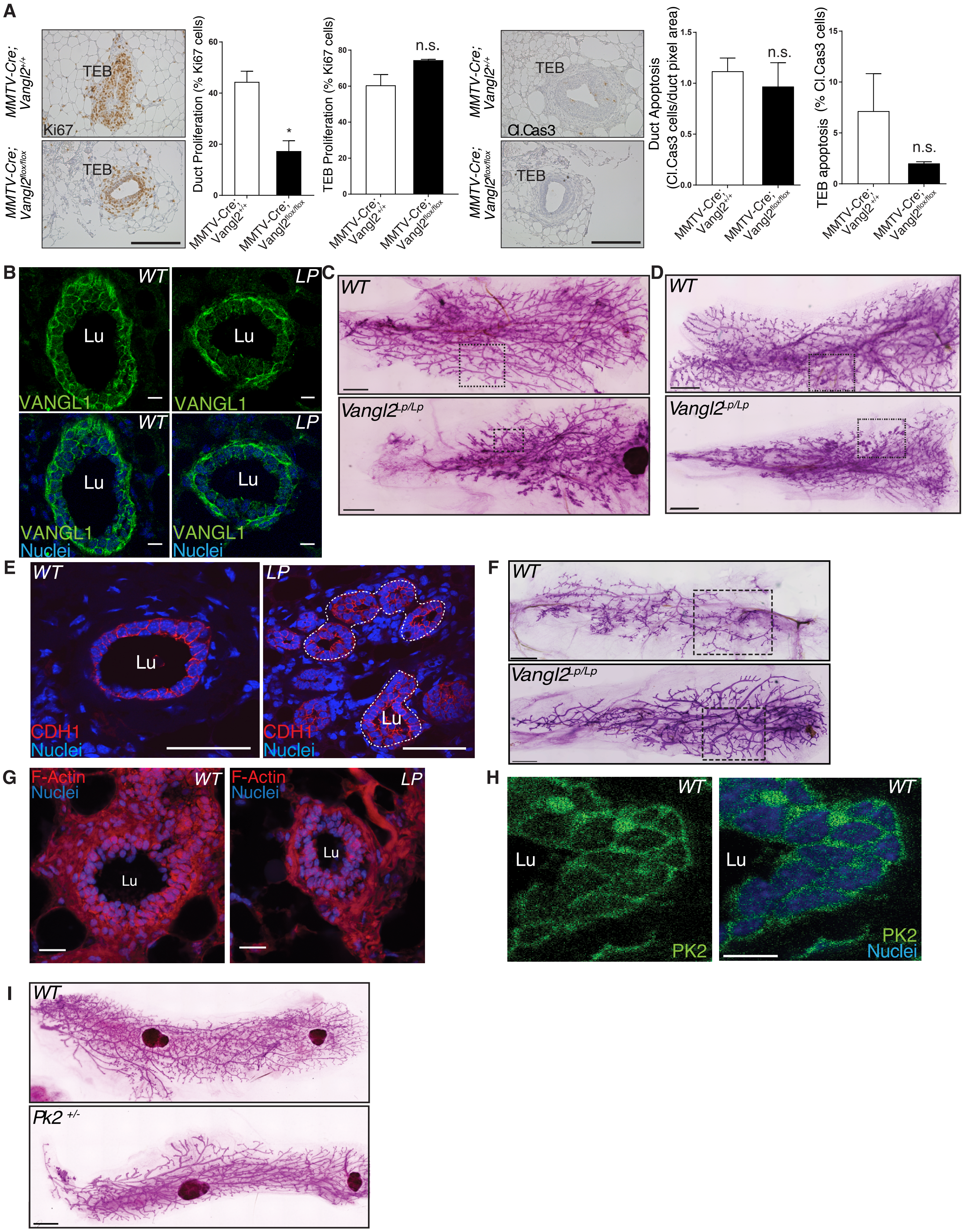
A
MMTV-Cre; Vangl2+/+
MMTV-Cre; Vangl2flox/flox
TEB
Ki67
Duct Proliferation (% Ki67 cells)
TEB Proliferation (% Ki67 cells)
n.s.
*
Cl.Cas3
Duct Apoptosis (Cl.Cas3 cells/duct pixel area)
TEB apoptosis (% Cl.Cas3 cells)
B
WT
LP
Lu
VANGL1
Nuclei
C
D
Vangl2Lp/Lp
E
CDH1
F
G
F-Actin
H
PK2
I
Pk2 +/-

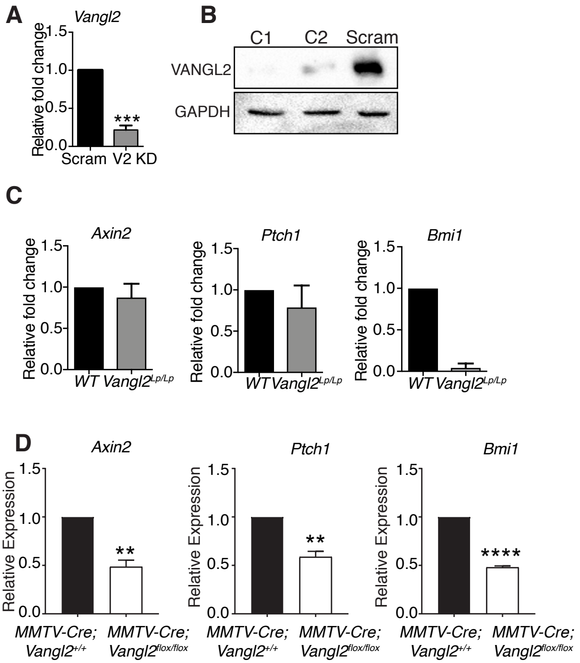

A
Vangl2
Relative fold change
1.5
1.0
0.5
0.0
***
Scram
V2 KD
B
C1
C2
Scram
VANGL2
GAPDH
C
Axin2
Ptch1
Bmi1
Relative fold change
WT
Vangl2Lp/Lp
D
Axin2
Ptch1
Bmi1
Relative Expression
**
**
****
MMTV-Cre; Vangl2+/+
MMTV-Cre; Vangl2flox/flox

Figure 1E

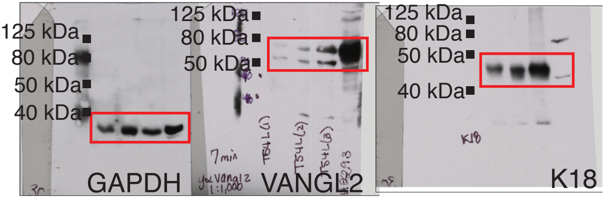

Sup. Figure 3B

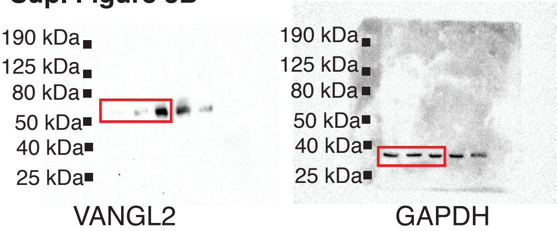

Gene	Forward	Reverse
Axin2	GCAGCTCAGCAAAAAGGGAAA T	TACATGGGGAGCACTGTCTCG T
Bmi1	CCAATGAAGACCGAGGAGAA	TTTCCGATCCAATCTGCTCT
Ptch1	ATGGGCCTCATTGGGATCAA	AGCATAGCCCTGTGGTTCTT
Vangl1	AAGCAAAGAGCGGATGTG	CGATGGCAAGGTAGTGGA
Vangl1	GATGCTGTTAGGAGGTTCGG	AGTCCCGCTTCTACAGCTTG
Vangl2	CCAGCCGCTTCTACAATGTC	TCTCCAGGATCCACACTGC
Vangl2	TGCTGGACAAGTGGGCTTAT	GTGCGCTGCGGATACAAA
GAPDH	TTCACCACCATGGAGAAGGC	CCCTTTTGGCTCCACCT
